# A Nationwide Study on the Prevalence of Peripheral Neuropathy in Patients with Type 2 Diabetes Mellitus in Greece—The PRENEDIG Study

**DOI:** 10.3390/jcm14196723

**Published:** 2025-09-23

**Authors:** Ilias N. Migdalis, Nikolaos K. Tentolouris, Triantafyllos P. Didangelos, Nikolaos Papanas, Magdalini X. Bristianou, Anastasia N. Mavrogiannaki, on behalf of the PRENEDIG Study

**Affiliations:** 1 Diabetic Clinic, Lefkos Stavros Hospital, 11528 Athens, Greece; 2 First Department of Propaedeutic Internal Medicine and Diabetes Centre, Medical School, National and Kapodistrian University of Athens, Laiko Hospital, 11527 Athens, Greece; ntentolouris@yahoo.gr; 3 First Department of Propaedeutic Internal Medicine and Diabetes Centre, Medical School, Aristotle University of Thessaloniki, Ahepa Hospital, 54636 Thessaloniki, Greece; didang@auth.gr; 4 Second Department of Internal Medicine and Diabetes Centre, Medical School, Democritus University of Thrace, Alexandroupolis Hospital, 68100 Alexandroupolis, Greece; papanasnikos@yahoo.gr; 5 Department of Internal Medicine and Diabetic Clinic, Lamia Hospital, 35100 Lamia, Greece; mbristianou@gmail.com; 6 Second Department of Internal Medicine and Diabetes Centre, NIMTS Hospital, 11521 Athens, Greece; anastasia_mavrogiannaki@yahoo.com

**Keywords:** type 2 diabetes mellitus, diabetic peripheral neuropathy, prevalence, risk factors, nationwide study, cross-sectional study, Greece, multivariate analysis, screening tools

## Abstract

**Background/Objectives:** Peripheral neuropathy (PN) is a common complication of diabetes mellitus (DM) with prevalence estimates showing considerable variation across studies. This study aimed to assess the prevalence and risk factors of PN in adult Greek subjects with type 2 diabetes mellitus (T2DM). **Methods:** Τhe PRENEDIG (PREvalence of peripheral NEuropathy in type 2 DIabetes in Greece) study was a nationwide, cross-sectional multicenter study based on data collected from hospital-based diabetes clinics and primary care practices from January 2024 to June 2024 in Greece. Diabetic peripheral neuropathy (DPN) prevalence and severity were evaluated using the Neuropathy Symptom Score (NSS) and the Neuropathy Disability Score (NDS). Additional sensory assessment tools were considered to support clinical evaluation. Multivariate regression analysis examined the association between DPN and potential risk factors. **Results:** Among the study population (n = 1807), the overall DPN prevalence was 18.87% and increased with longer diabetes duration. DPN prevalence among participants with over 10 years of T2DM reached 26.49%. Logistic regression analysis identified several independent predictors of DPN including diabetes duration > 10 years (*p* < 0.001), arterial hypertension in participants with diabetes duration < 10 years (OR = 2.69, CI: 1.68–4.30, *p* < 0.001), HbA1c levels (OR = 1.20, CI: 1.10–1.31, *p* < 0.001), and age (OR = 1.02, CI: 1.00–1.03, *p* = 0.024). An interaction-related association was observed, with arterial hypertension not increasing the risk of DPN any further in participants with disease duration > 10 years (OR: 3.73 vs. 3.80 with or without arterial hypertension, respectively). Sensory assessment tools further validated DPN diagnosis. **Conclusions:** In Greece, DPN is a common complication, affecting nearly one in five T2DM patients. The results of the study reinforce the importance of routine screening, particularly among older patients and those with longer diabetes duration to facilitate early detection and timely management of DPN and its associated complications.

## 1. Introduction

Diabetes mellitus (DM) represents a growing public health challenge globally. According to estimates from the International Diabetes Federation (IDF), approximately 589 million adults aged 20–79 years currently live with diabetes, corresponding to almost 1 in 9 adults. This number is projected to rise to 853 million by 2050, affecting more than one in 10 adults. In Greece, the first national Health Examination Survey (HES) conducted during the period 2013–2016 estimated the prevalence of diabetes at 11.9% [1], with type 2 diabetes mellitus (T2DM) comprising approximately 95% of all diagnosed diabetes cases and reflecting the global distribution pattern [2,3]. More recent estimates from the IDF indicate that in 2024, the prevalence of diabetes among adults aged 20–79 in Greece was 11.4%, with an age-standardized rate of 8% [4].

T2DM carries a significant economic and social burden, as it is currently the 10th leading cause of disease burden globally in terms of deaths, and is estimated to become the 9th leading cause by 2050 [5,6]. The sharp rise in prevalence driven by aging populations and lifestyle changes is anticipated to be accompanied by a proportional increase in the incidence of diabetes-associated complications [4,6,7]. Among these, diabetic peripheral neuropathy (DPN) is the most common long-term complication leading to significant morbidity and substantially reducing quality of life [8,9,10,11].

DPN is the most prevalent form of diabetic nerve damage, typically presenting as distal symmetric polyneuropathy, with sensory loss beginning in the lower limbs and progressing proximally [12]. DPN is characterized by impaired nerve conduction velocity and abnormal sensory function, which result from nerve demyelination, axonal atrophy, and other forms of nerve damage [12,13,14].

The pathogenesis of diabetic peripheral neuropathy (DPN) is complex and multifactorial, involving metabolic, vascular, and inflammatory mechanisms [15,16]. Chronic hyperglycemia is a central driver, activating the polyol and protein kinase C pathways and promoting the accumulation of advanced glycation end products, which trigger oxidative stress and neuronal injury [12,17,18,19]. Dyslipidemia, obesity, insulin resistance, and impaired insulin signaling further exacerbate mitochondrial dysfunction, oxidative damage, and inflammatory responses, ultimately leading to neuronal and glial cell impairment [12]. Microvascular abnormalities, including endothelial dysfunction and tissue hypoxia, reduce neurotrophic support and aggravate axonal injury [20,21]. In addition, chronic low-grade inflammation, reflected by elevated cytokines such as TNF-α, IL-6, and ICAM-1, and inflammatory markers including NLR, PLR, and SII, has been strongly associated with the development and progression of DPN [22,23,24].

Clinically, DPN contributes to impaired gait, falls, chronic neuropathic pain, and depression [8,25]. It is a major risk factor for diabetic foot ulcers, which frequently result in non-traumatic lower-limb amputations and also contributes to increased all-cause and cardiovascular mortality [9,26]. Beyond its clinical consequences, DPN places a significant economic burden on healthcare systems through direct medical costs and on society through lost productivity due to disability [27].

Despite its severity, DPN remains less studied compared with other diabetic complications [28,29,30,31], highlighting the importance of early detection and risk identification to prevent DPN complications [32,33,34,35]. Estimates of DPN prevalence show considerable variation depending on patient population, applied diagnostic criteria and ethnic differences [36,37,38,39,40,41,42,43]. To our knowledge, there are no published data on the prevalence of DPN among patients with T2DM in Greece. Therefore, the aim of this nationwide, multicenter study was to examine the prevalence of peripheral neuropathy and its associated risk factors among adult Greek T2DM patients.

## 2. Materials and Methods

### 2.1. Study Design

Τhe PRENEDIG (PREvalence of peripheral NEuropathy in type 2 DIabetes in Greece) study was a national, non-interventional, cross-sectional, multicenter study. Participants were recruited between January 2024 and June 2024 and a single clinical visit was performed per participant. The study was approved by all participating ethics committees and informed consent was obtained from all study participants. The study was conducted at hospital-based diabetes clinics and primary care practices from various regions across Greece, selected to reflect clinical practice variations and provide a representative sample of the Greek population. The data was collected simultaneously in all centers over a 6-month period.

### 2.2. Study Population

Eligible for participation were men and women aged 18 years and older with a diagnosis of T2DM and recent (within the last 6 months) laboratory data for the following: cholesterol, triglycerides, high-density lipoprotein cholesterol (HDL-C), low-density lipoprotein cholesterol (LDL-C), glucose, HbA1c, urea, creatinine, complete blood count exam, vitamin B12, and urine albumin/creatinine ratio (ACR, calculated from a morning urine sample or a random urine sample), Glomerular Filtration Rate (GFR, CKD-EPI 2021 equation). Participants with type 1 diabetes mellitus, as well as history of insulin initiation during the first 3 years after diagnosis of T2DM or within 2 months prior to study initiation were excluded.

### 2.3. Clinical and Demographic Data

Demographic and clinical characteristics, along with the participant’s diabetes-related history, were recorded. Participant data including weight, height, and blood pressure were measured during the study visit. Age, gender, diabetes duration, treatment for DPN or other conditions, and history of arterial hypertension, cerebrovascular events, foot ulcers, heart failure, dyslipidemia and smoking were collected. Diabetic macrovascular (coronary artery disease, peripheral artery disease) and microvascular disease (retinopathy, chronic kidney disease) were also documented, based on participants’ record files. Obesity was defined as body mass index (BMI) > 30 kg/m^2^. The laboratory parameters recorded included total cholesterol, triglycerides, low-density lipoprotein cholesterol (LDL-C), high-density lipoprotein cholesterol (HDL-C), glucose, hemoglobin A1c (HbA1c), urea, creatinine, white blood cell (WBC) count, WBC differential count, hematocrit (Hct), hemoglobin (Hb), platelet count (PLT), vitamin B12, and albumin-to-creatinine ratio (ACR).

### 2.4. Study Objectives

The primary objective of the study was to assess the prevalence of DPN based on the Neuropathy Disability Score (NDS) and Neuropathy Symptom Score (NSS) in participants with T2DM followed in hospital-based diabetes clinics and primary care practices across Greece. Secondary objectives included the recording of clinical and sociodemographic characteristics of participants with T2DM and DPN as well as the association between DPN and participant-related factors such as sex, smoking status, chronic diabetes complications, comorbidities, and loss of protective sensation.

### 2.5. Assessment of Diabetic Peripheral Neuropathy

The neuropathy disability score (NDS), neuropathy symptom score (NSS) and biothesiometer test (Bio Medical Instrument Co., Newbury, OH, USA) for each participant were derived as described below, based on the methodology proposed by Young et al. [44]. The diagnosis of peripheral neuropathy required, at a minimum, neuropathy disability score with at least moderate signs of disease severity (NDS ≥ 6) with or without symptoms or mild signs of disease severity with moderate symptoms (NDS ≥ 3 and NSS ≥ 5). Only mild signs or mild symptoms were not considered adequate to make the diagnosis of peripheral neuropathy.

#### 2.5.1. Νeuropathy Scores

The NSS was based on participant-reported leg symptoms, including type (e.g., burning, numbness), location (feet, calves, thighs), timing (day/night), sleep disruption, and alleviating factors, with a maximum score of 9; scores ≥ 3 indicated neuropathic symptoms. The NDS involved clinical evaluation of ankle reflexes and sensory perception (vibration, pin-prick, and temperature) at the great toe, with a maximum score of 10; scores ≥ 3 indicated neuropathic signs. Both scales categorized severity as mild, moderate, or severe based on score ranges.

#### 2.5.2. Monofilament Testing

Sensory testing using a 5.07 Semmes–Weinstein monofilament (Bailey Instruments, Manchester, UK) was conducted at three sites on each foot, specifically the plantar surface of the great toe, and the heads of the 1st and 3rd metatarsals [45]. Each site was tested three times, including one simulated (sham) application, to assess response consistency. Testing was not performed over callused areas. A site was considered to have normal sensation if the participant correctly identified at least two out of three applications. The test was classified as abnormal and indicative of loss of protective sensation if the response was incorrect at any one of the tested sites.

#### 2.5.3. Biothesiometer Test

In the centers equipped with a biothesiometer (Bio Medical Instrument Co., Newbury, OH, USA), the vibration perception threshold (VPT), which indicates the minimal intensity of vibration that a person can perceive, was assessed at the great toe concurrently with the calculation of the neuropathy disability score. The device was positioned vertically on the pulp of the great toe to evaluate vibration sensitivity, and the final VPT value for each participant was based on the average of three separate measurements.

### 2.6. Statistical Analysis

Statistical analyses were performed using R software (version 4.4.3, Foundation for Statistical Computing, Vienna, Austria). Quantitative variables were based on the number of observations (n) in each group and described using appropriate measures of central tendency (mean, median) and dispersion (standard deviation, minimum, maximum). The crude DPN percentages in the study population, as assessed by the NDS and/or NSS scales, were compared across subgroups of interest (gender, smoking status, diabetic complications, comorbidities, and loss of protective sensation) using the chi-squared (χ^2^) statistic. Group comparisons for age, body mass index, diabetes duration, laboratory values, and biothesiometer test results, based on the presence or absence of DPN, were conducted using the t distribution. A multivariate stepwise logistic regression model based on Akaike Information Criterion was applied to identify independent predictors of DPN, including demographic, social, clinical variables, and treatment factors. The strength of associations was expressed as odds ratios (ORs) with 95% confidence intervals. All tests 2-sided and level of statistical significance was set at α = 0.05.

## 3. Results

### 3.1. Participant Demographic and Clinical Characteristics

A total of 1807 participants were enrolled in the study from 30 centers. The mean age was 65 years and 55.40% of the participants were male. Most participants belonged to the age groups 60 to 69 years (36.47%) and 70 to 79 years (28.06%), together comprising over 64% of the total cohort. Consistent with the diagnosis of T2DM, mean body mass index (BMI) was 30.67 kg/m^2^ with 37.69% of the population being overweight and 47.92% being obese. The duration of type 2 diabetes mellitus (T2DM) was <5 years in 32.37% of participants, 5–10 years in 28.56%, and >10 years in 39.07%. Most participants had comorbid hypertension (71.50%), dyslipidemia (85.50%), and/or diabetes-related chronic microvascular and macrovascular complications, with coronary artery disease (CAD, 19.37%) and chronic kidney disease (CKD, 14.83%) being the most common ones.

Patients in the DPN subpopulation (N = 341) shared similar characteristics with the overall study population, although a higher proportion had a longer duration of diabetes, with 54.84% having diabetes for more than 10 years. The mean (SD) age was 68.02 (10.304) years, the mean (SD) BMI was 30.45 (5.617) kg/m^2^ and the mean (SD) vitamin B12 was 387.68 (164.229) pg/mL. The age distribution of DPN patients showed that only 5.3% were younger than 50, while the majority (66%) were between 60 and 79 years old. Additionally, complications such as retinopathy, PAD, CKD, and foot ulcers were more prevalent in the DPN group (Table 1).

### 3.2. DPN Prevalence and Associated Risk Factors

**Figure 1:**
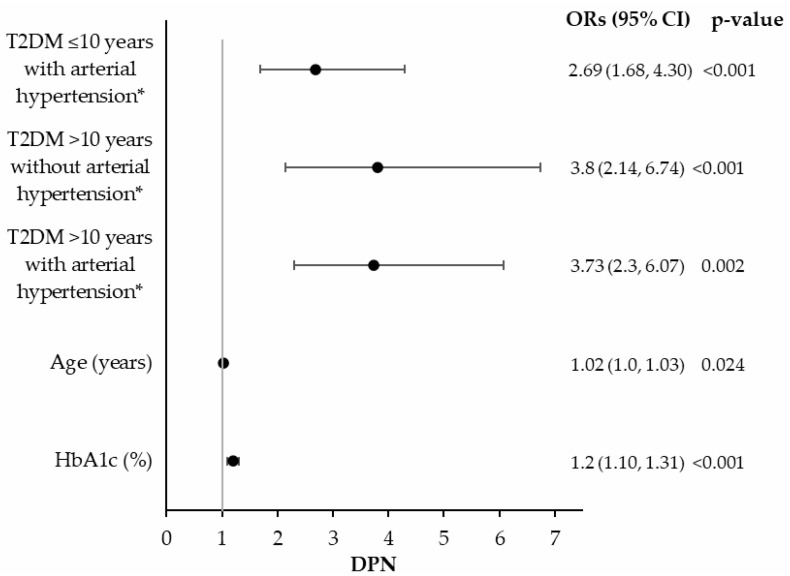
Forest plots of the associations between DPN prevalence and baseline characteristics. * Reference group: T2DM ≤ 10 years without arterial hypertension. T2DM: Type 2 diabetes mellitus, DPN: Diabetic peripheral neuropathy, HbA1c: Hemoglobin A1c, OR: Odds ratio, CI: Confidence interval.

The crude prevalence of DPN in the full analysis set was 18.87% (95% CI: 17.09–20.75%), similar in both male and female participants. Among affected participants, 17.89% had diabetes for less than 5 years, 27.27% for 5–10 years and 54.84% for more than 10 years (Table 1). A logistic regression model was used to estimate independent predictive factors for DPN prevalence that included diabetes duration, arterial hypertension, HbA1c, Hct, age, and urea. Diabetes-related complications were not included in the model because they were underrepresented in the studied population. Results showed that the duration of diabetes for more than 10 years and the presence of arterial hypertension were statistically significant independent predictive factors for DPN diagnosis. Notably, an interaction-related association was observed between diabetes duration exceeding 10 years and arterial hypertension resulting in a lower risk when both factors were present than anticipated based on their individual risks. Participants having T2DM for more than 10 years without arterial hypertension had similar odds for DPN (OR = 3.80, 95% CI: 2.14–6.74, *p* < 0.001) to those exhibiting both conditions (OR = 3.73, 95% CI: 2.30–6.07, *p* = 0.002) (Figure 1). Furthermore, every additional year of age increased the odds of DPN occurrence by 2% (OR = 1.02, 95% CI: 1.00–1.03, *p* = 0.024). DPN prevalence was also significantly associated with elevated HbA1c (OR = 1.20, 95% CI: 1.10–1.31, *p* < 0.001).

### 3.3. Assessment of Sensory Function

#### 3.3.1. Semmes–Weinstein 5.07 Monofilament Test

Sensory testing using a 5.07 Semmes–Weinstein monofilament was performed in 1700 subjects and revealed a significant correlation (*p* < 0.001) between loss of protective sensation and the presence of DPN. Specifically, 55.35% of participants with DPN that were evaluated for loss of protective sensation showed loss of protective sensation, compared to only 6.08% of those with normal neural function. The loss of protective sensation increased with longer diabetes duration, as 8.70% of T2DM patients with less than 5 years of disease duration had loss of sensation, while 14.11% of participants with T2DM duration of 5–10 years and 21.70% of participants with more than 10 years of diabetes experienced loss of protective sensation (Table 2).

#### 3.3.2. Biothesiometer Test

A total of 629 individuals underwent the biothesiometer test to further assess DPN by measuring the vibration perception threshold (VPT) at the great toe, an indicator of loss of protective sensation. Results showed that the VPT in DPN patients was significantly higher compared to participants without DPN both in the right and left great toe (*p* < 0.001). The VPT of the right and left great toe increased with longer diabetes duration (Table 3).

## 4. Discussion

Diabetic peripheral neuropathy (DPN) is a global health concern affecting both developed and developing countries. However, efforts to systematically monitor its global prevalence are limited, as multi-country studies using standardized protocols are sparse and have notable limitations. Estimates of DPN prevalence are affected by various factors, including the diagnostic methods and criteria used, as well as characteristics of the type 2 diabetes (T2D) population, such as age, duration of diabetes, and the presence of diabetes-related or other comorbidities [46]. Nonetheless, most high-quality studies report that DPN affects approximately 20–50% of individuals with T2DM [12,14,43,46,47].

In the present study, using NDS/NSS as a diagnostic tool, we observed a DPN prevalence of 18.9% among participants with T2DM in Greece, which falls within the lowest end of the range reported by European studies [41,44,48,49,50,51]. The comparatively low prevalence reported by our study may be partially attributed to the diagnostic methods employed. The NSS and NDS (symptom and sign scores) have shown strong overall diagnostic performance in detecting DPN, especially when used together, and represent the most commonly used diagnostic tool in clinical practice in Greece. Nevertheless, several studies have employed the MNSI diagnostic tool, which is another reliable method for the diagnosis of DPN [43,52]. A recent meta-analysis of 29 studies with 50,112 diabetic participants showed that studies employing NSS and NDS reported lower DPN prevalence (21.5%, 95%CI 9.6–33.3) compared to those using MNSI (27.8%, 95% CI 18.9–36.6), with the latter being the most commonly used diagnostic tool [43].

Despite the lower overall prevalence observed in our cohort, we found similar risk factors associated with the presence of DPN in T2DM patients. In our model the strongest predictor for the presence of DPN was the duration of the disease, with duration > 10 years being significantly associated with higher probability for the presence of DPN. Also, DPN was more prevalent with increasing age with every additional year of age increasing the odds of DPN occurrence by 2%. These results are in line with the results of previous studies supporting that the burden of diabetic peripheral neuropathy is higher in older age and among adults with long-standing diabetes [41,44,48,53,54,55].

The multivariate regression model identified elevated HbA1c as another independent risk factor for DPN (OR = 1.20, 95% CI: 1.10–1.31), despite the fact that the diabetic patients included in the study were relatively well-controlled, with a mean HbA1c of 7.0% [56]. Elevated HbA1c has long been considered closely associated with DPN in T2DM [57,58,59,60] and the development of DPN in the study population suggests that a lower HbA1c value may be necessary to prevent the onset and progression of neuropathy.

Our study has also identified an association between DPN and arterial hypertension, in line with other published studies [61,62]. Nevertheless, when arterial hypertension as a risk factor was present in participants with a disease duration > 10 years, it was shown not to increase the risk of DPN any further (OR: 3.73 for disease duration > 10 years and arterial hypertension vs. OR: 3.80 for disease duration > 10 years without/unknown presence of arterial hypertension). This finding highlights that the long duration of the disease can be identified as the strongest predictor for the presence of DPN in participants with T2DM.

Comorbid microvascular and macrovascular complications have also been identified as a factor predisposing to neuropathy development [14,41,49,63,64,65,66,67,68]. Among these, kidney disease shows a particularly strong and well-established association with peripheral neuropathy [66,67,69,70]. This is attributed to combined pathological effects, with CKD-driven mechanisms such as potassium-related axonal dysfunction [71,72,73] and diabetes-related factors like insulin resistance further contributing to nerve injury [17,74].

The results of the study showed a higher prevalence of chronic, diabetes-related complications among participants with DPN including foot ulcers, peripheral arterial disease (PAD), arterial hypertension, retinopathy, and chronic kidney disease (CKD), indicating a potential association. Nevertheless, the inclusion of such diabetes-related complications in the logistic regression analysis was not achieved due to the fact that they were underrepresented in the studied population.

Patients with DPN may experience loss of protective sensation in their feet leading to potential injuries that pose significant risk of ulceration and ultimately amputation [75,76,77]. In our study, sensory testing using a 5.07 Semmes–Weinstein monofilament and the biothesiometer test (where available) confirmed the significant correlation (*p* < 0.001) between loss of protective sensation and the presence of DPN. Loss of protective sensation increased with longer diabetes duration, highlighting the need for early diagnosis and management of DPN in order to avoid such complications.

Studies have identified an association between vitamin B12 deficiency and DPN [78]. Vitamin B12 deficiency itself has also been shown to have neurological consequences, including peripheral neuropathy. In our study, vitamin B12 levels were normal (>200 pg/mL equivalent to >148 pmol/L) [79,80] and similar between cohorts. Nevertheless, diabetic patients over 60 years old may exhibit signs of neurological dysfunction, including numbness, tingling, and muscle weakness, even when their B12 levels are above the standard threshold. According to recent suggestions, B12 levels between 150 and 400 pmol/L, such as those observed in our study, should be classified as a “relative” deficiency in diabetic individuals [81] and might benefit from B12 supplementation [82].

The present study is the first to evaluate the prevalence of DPN in Greece. It has several strengths, including its nationwide, real-world approach, the large sample included, and the ethnic homogeneity of the population studied. Moreover, participants were recruited from both hospital and primary care settings, encompassing a broad range of disease severity and age, which enhances the representativeness of the sample and helps to minimize selection bias. Although the large sample size improves the accuracy of study results, it also led to statistically significant differences between patients with or without DPN in the univariate analysis that were of limited clinical relevance. This further underscores the importance of the multivariate regression analysis, which allowed adjustment for confounding and identification of independent associations. Nonetheless, certain limitations should be acknowledged. Assessment of DPN was conducted solely using the NSS and NDS scales, without additional clinical diagnostic tools (e.g., EMG), as such tools are not commonly available in primary care settings in Greece. Accordingly, comorbidities including diabetic complications were documented as either present or absent based exclusively on existing medical records, without supplementary clinical evaluation. This approach is widely adopted in epidemiological studies [83,84,85] and, to our knowledge, no publications have compared it directly with clinical or laboratory confirmation. Finally, the cross-sectional nature of the study restricts the ability to draw causal inferences.

## 5. Conclusions

The prevalence of DPN among Greek T2DM patients is 18.9%. T2DM duration, increasing age, and elevated HbA1c were identified as the most important risk factors in the Greek population. Our analysis involving the investigation of potential interactions has also allowed the identification of the long T2DM duration as the strongest predictor for the presence of DPN in patients with T2DM. Our results reinforce the importance of routine screening, particularly among older patients and those with longer diabetes duration, but also among patients with shorter diabetes duration complicated by the presence of arterial hypertension, to facilitate early detection and timely management of DPN and its associated complications.

## Figures and Tables

**Table 1:** Sociodemographic, clinical, and laboratory characteristics of participants by presence of DPN.

Characteristic	Class	Total, n = 1807	T2DM Patients with DPN, n = 341	T2DM Patients Without DPN, n = 1466
Sex	(males), n (%)	1001 (55.40%)	194 (56.89%)	807 (55.05%)
	(females), n (%)	806 (44.60%)	147 (43.11%)	659 (44.95%)
Age *	(years) mean (SD)	65.26 (10.390)	68.02 (10.304)	64.62 (10.308)
	(years), n (%)			
	<50	131 (7.25%)	18 (5.28%)	113 (7.71%)
	50–59	379 (20.97%)	54 (15.84%)	325 (22.17%)
	60–69	659 (36.47%)	121 (35.48%)	538 (36.70%)
	70–79	507 (28.06%)	104 (30.50%)	403 (27.49%)
	≥80	131 (7.25%)	44 (12.90%)	87 (5.93%)
Anthropometric characteristics	Weight (kg)	86.67 (19.133)	85.98 (19.341)	86.83 (19.088)
	BMI (kg/m^2^) mean (SD)	30.67 (6.088)	30.45 (5.617)	30.72 (6.194)
	Overweight (BMI:25–29,9), n (%)	681 (37.69%)	125 (36.66%)	556 (37.95%)
	Obese (BMI ≥ 30), n (%)	866 (47.92%)	163 (47.80%)	703 (47.99%)
Smoking, n (%)	Never	986 (54.57%)	183 (53.67%)	803 (54.77%)
	Former	485 (26.84%)	98 (28.74%)	387 (26.40%)
	Current	336 (18.59%)	60 (17.60%)	276 (18.83%)
Diabetes duration, n (%) *	<5 years	585 (32.37%)	61 (17.89%)	524 (35.74%)
	5–10 years	516 (28.56%)	93 (27.27%)	423 (28.85%)
	>10 years	706 (39.07%)	187 (54.84%)	519 (35.40%)
Comorbidities	Dyslipidemia, n (%)	1545 (85.50%)	297 (87.87%)	1248 (85.36%)
	Heart failure, n (%) *	115 (6.36%)	37 (11.49%)	78 (5.62%)
	Foot ulcers, n (%) *	78 (4.32%)	48 (14.08%)	30 (2.05%)
	Arterial hypertension, n (%) *	1292 (71.50%)	279 (81.82%)	1013 (69.72%)
Macrovascular complications	Coronary artery disease, n (%) *	350 (19.37%)	79 (24.09%)	271 (18.90%)
	Peripheral arterial disease, n (%) *	115 (6.36%)	52 (16.94%)	63 (4.73%)
	Cerebrovascular events, n (%)	94 (5.20%)	21 (6.23%)	73 (5.00%)
Microvascular complications	Retinopathy, n (%) *	164 (9.08%)	76 (30.52%)	88 (7.18%)
	Chronic kidney disease, n (%) *	268 (14.83%)	90 (26.87%)	178 (12.43%)
Laboratory test results	Total cholesterol (mg/dL) mean (SD)	157.91 (40.368)	157.08 (43.256)	158.10 (39.680)
	Triglycerides (mg/dL) mean (SD)	140.01 (73.296)	142.21 (74.900)	139.50 (72.938)
	LDL-C (mg/dL) mean (SD)	83.69 (34.975)	83.95 (38.612)	83.62 (34.089)
	HDL-C (mg/dL) mean (SD)	46.59 (11.938)	45.90 (11.397)	46.75 (12.058)
	Glucose (mg/dL) mean (SD)	132.94 (43.149)	137.57 (54.812)	131.84 (39.849)
	HbA1c (%) mean (SD) *	6.95 (1.340)	7.26 (1.607)	6.87 (1.258)
	Urea (mg/dL) mean (SD)*	39.82 (16.027)	43.80 (19.273)	38.88 (15.008)
	Creatinine (mg/dL) mean (SD) *	0.92 (0.349)	0.98 (0.427)	0.91 (0.326)
	WBC (10^3^/L) mean (SD)	7663.68 (1958.057)	7718.39 (2023.456)	7650.63 (1942.643)
	WBC differential count (%) mean (SD) *	51.53 (20.028)	49.08 (22.658)	52.11 (19.315)
	Hct (%) mean (SD) *	41.90 (4.268)	41.05 (4.602)	42.11 (4.160)
	Hb (g/dL) mean (SD) *	13.75 (1.556)	13.50 (1.787)	13.82 (1.488)
	PLT (10^3^/L) mean (SD)	247,337.30 (63,784.816)	249,642.86 (67,737.075)	246,794.22 (62,831.376)
	Vitamin B12 (pg/mL) mean (SD) *	365.56 (149.275)	387.68 (164.229)	360.55 (145.282)
	ACR (mg/g) mean (SD) *	65.12 (246.191)	140.02 (434.844)	48.09 (173.094)

BMI: Body Mass Index, LDL-C: Low Density Lipoprotein–Cholesterol, HDL-C: High-Density Lipoprotein–Cholesterol, HbA1c: Hemoglobin A1c, WBC: White Blood Cell count, Hb: Hemoglobin, PLT: Platelet count, ACR: Albumin-to-Creatinine Ratio. Variables marked with an asterisk (*) indicate statistical significance at *p* < 0.05.

**Table 2:** Results of the Semmes–Weinstein 5.07 monofilament test by T2DM duration and by DPN presence.

	Total, n = 1700	T2DM < 5 Years, n = 552	T2DM 5–10 Years, n = 489	T2DM > 10 Years, n = 659	T2DM Patients with DPN, n = 318	T2DM Patients Without DPN, n = 1382
Loss of protective sensation, n (%)						
Yes	260 (15.29%)	48 (8.70%)	69 (14.11%)	143 (21.70%)	176 (55.35%)	84 (6.08%)
No	1440 (84.71%)	504 (91.30%)	420 (85.89%)	516 (78.30%)	142 (44.65%)	1298 (93.92%)

T2DM: Type 2 diabetes mellitus, DPN: Diabetic peripheral neuropathy.

**Table 3:** Results of the Biothesiometer test by T2DM duration and by DPN presence.

	Total, n = 629	T2DM < 5 Years, n = 183	T2DM 5–10 Years, n = 171	T2DM > 10 years, n = 275	T2DM Patients with DPN, n = 125	T2DM Patients Without DPN, n = 504
VPT (volts) mean (SD)						
Right toe	16.72 (10.613)	13.63 (7.595)	17.38 (12.011)	18.36 (11.001)	26.59 (13.744)	14.27 (7.990)
Left toe	16.92 (10.693)	14.33 (8.392)	17.59 (12.369)	18.24 (10.672)	26.93 (13.760)	14.44 (8.066)

T2DM: Type 2 diabetes mellitus, DPN: Diabetic peripheral neuropathy, VPT: Vibration perception threshold.

## Data Availability

The data presented in this study is available upon request from the corresponding author.

## Abbreviations

The following abbreviations are used in this manuscript:

PN	Peripheral neuropathy
DM	Diabetes mellitus
T2DM	Type 2 diabetes mellitus
DPN	Diabetic peripheral neuropathy
NSS	Neuropathy Symptom Score
NDS	Neuropathy Disability Score
IDF	International Diabetes Federation
HES	Health Examination Survey
HDL-C	High-density lipoprotein cholesterol
LDL-C	Low-density lipoprotein cholesterol
ACR	Albumin-to-creatinine ratio
GFR	Glomerular Filtration Rate
BMI	Body mass index
HbA1c	Hemoglobin A1c
WBC	White blood cell count
Hct	Hematocrit
Hb	Hemoglobin
PLT	Platelet count
VPT	Vibration perception threshold
OR	Odds ratio
CKD	Chronic kidney disease
PAD	Peripheral artery disease
SD	Standard deviation
MNSI	Michigan Neuropathy Screening Instrument
